# Knowledge Related to Hypertension Risk Factors, Diet, and Lifestyle Modification: A Comparative Study Between Hypertensive and Non-Hypertensive Individuals

**DOI:** 10.7759/cureus.9890

**Published:** 2020-08-20

**Authors:** Amira A Asiri, Sulaiman Asiri, Hanan Asiri

**Affiliations:** 1 Internal Medicine, King Khalid University, Abha, SAU; 2 General Surgery, Asir Central Hospital, Abha, SAU; 3 Quality Improvement & Patient Safety, Armed Forces Hospitals Southern Region, Khamis Mushayt, SAU

**Keywords:** hypertension, risk factors, diet, lifestyle, modifications.

## Abstract

Introduction

This study was conducted to explore the three-dimensioned knowledge level of hypertension risk factors (i.e. a three parts questionnaire in addition to the demographic section that discusses in each a dimension of hypertension-related knowledge which is hypertension high-risk factors dimension, diet modifications dimension, and lifestyle behavior modifications dimension to either prevent or control hypertension), the needed diet and lifestyle modifications to either cope with or prevent hypertension among the study participants. The study also examined the significance of the relationship between the two groups and their knowledge of hypertension three dimensions as well as their age, gender, family history, education, and participants’ occupation.

Methodology

In this cross-sectional study, a disproportionate stratified random sampling was used which stratified the sample into two groups i.e. hypertensive and non-hypertensive individuals between 30 and 50 years old from the community of the city of Abha, Saudi Arabia. A developed modified three-dimensioned self-administered online questionnaire^ ^was used which was tested afterward for reliability and validity. For this study, the sample size is 384 while the response rate achieved is 60.4% where the data was collected within a timeframe of two weeks.

Results

The respondents had a high level of knowledge regarding the risk factors, diet modifications, and lifestyle modifications. Both groups have the same knowledge level that does not differ significantly. Gender is not a factor of significance for hypertension, but a family history of hypertension shows a significant relationship among the two groups. Age, education, and occupation do not relate significantly among both groups.

Conclusion

The results might be contributed to the participants' high educational level as well as the fact that a lot of them have a family history of hypertension.

## Introduction

One of the most common health conditions in the world today is hypertension or high blood pressure, which is considered as one of the leading risk factors for death [1]. In our literature review, we found out that in Saudi Arabia, most studies were concerned about studying the prevalence of hypertension. In a 2017 statistics report by the Saudi Ministry of Health (MOH), it was concluded that hypertension has a high prevalence among Saudi people who are 55 years old and above (51.2% among people aged 55-64 years and up to 70% among people aged 65 years and older) [2].

Furthermore, in the study of Aldiab and his colleagues (2018), it was shown that hypertension and prehypertension are prevalent particularly among adult males. The prevalence of hypertension was 6.0% in males, 4.2% in females, and 4.9% in all subjects [3]. Another study was conducted by Alsaghah et al. (2019), which indicated that the total prevalence of hypertension among the studied Saudi population was 8.9%, with a number of factors such as increased age, female sex, overweight, obesity, diabetes mellitus (DM), and morbid obesity which were found to be significantly associated with developing hypertension [4].

In our view in the literature, we noticed that most studies in Saudi Arabia that were concerned with hypertension were mainly focused on studying the prevalence in patients diagnosed with hypertension. Hypertension prevalence studies concluded that it has a high prevalence among Saudi people [5]. In terms of the hypertension perception in non-hypertensive individuals, no studies as far to our knowledge and research have been conducted in Saudi Arabia that examines this topic in healthy individuals. Yet, we found studies that investigated the hypertension-prevention behavior among Saudi people which indicated the need for a national plan to prevent and control hypertension in Saudi Arabia [5].

Abd El-Hay and colleagues (2015) concluded that among their study participants, there is a “poor level of knowledge about hypertension and perceptions toward lifestyle-modification” [6]. Since that diet-related factors and lifestyle are usually considered modifiable, Abd El-Hay et al. indicated that perceptions of their effects have particular importance in controlling hypertension [6]. Similarly, Kusuma's (2009) study showed that the individual’s perception of hypertension plays an important role in changing their lifestyle behavior in order to prevent developing hypertension [7]. Aung and colleagues (2012) research revealed that some hypertension risk factors are modifiable such as diet, smoking, and overweight, while some are not modifiable, such as genetic predisposition and old age. For this reason, changing those modifiable risk factors may result in a decreased burden of hypertension, and people need to know that they are susceptible to develop hypertension to be able to start voluntary modifications in their lifestyle [8].

In addition, hypertension is a major factor of early death and cardiovascular disability that causes a huge economic burden to both the human capital loss and medical cost [9,10]. Therefore, preventing the occurrence of hypertension has been recommended to avoid such expenses in terms of human and financial losses [6]. Accordingly, studying the hypertension-related perception in non-hypertensive individuals in addition to the hypertensive patients’ in terms of their own level of knowledge and behavior regarding risk factors, diet, and lifestyle modifications that may prevent them from developing hypertension or control it if already developed is important and which this study aims at discovering. 

For this reason, we focused in our study on both the hypertensive and non-hypertensive population and on examining their perception and knowledge level as a first step that can help in further research to either prevent developing hypertension or control it once developed. Also, the study explored the significance of the relationship between the two groups and their level of knowledge of the risk factors, diet modifications, and lifestyle modifications as well as their age, gender, family history, education, and participants’ occupation.

## Materials and methods

This is a cross-sectional study where disproportionate stratified random sampling was used to recruit participants via social media online questionnaire from the community of the city of Abha, Saudi Arabia over a period of two weeks to ensure that each member of the target population had an equal probability of being selected into the sample i.e. both Saudi females and males aged between 30 and 50 years old and whether they were hypertensive or not. The sample was stratified into two groups i.e. hypertensive and non-hypertensive individuals. A developed self-administered online questionnaire that assesses the perception of participants’ knowledge of hypertension risk factors and the needed diet and lifestyle-related health behavior modifications that either prevent or help control hypertension was distributed. A developed modified three-dimensioned self-administered online questionnaire that was originally adapted from Abd El-Hay and El Mezayen's study [6] was used which was tested afterward for reliability and validity. This questionnaire has three dimensions in addition to the first demographic data section, namely, hypertension high-risk factors dimension, diet modifications dimension, and lifestyle behavior modifications dimensions to either prevent or control hypertension.

Moreover, the data for this study was collected from the community of the city of Abha, Saudi Arabia, which has a population of 236,157 as per the latest statistics available [11]. The sample size for this study is 384 participants, where subjects included both Saudi females and males aged between 30 and 50 years old whether they were hypertensive or not. We achieved a response rate of 60.4%. The data were collected within two weeks timeframe in the month of August 2019. A pilot study was conducted to verify the validity and reliability of the study data collection tool. The tool of the study consists of four sections i.e. general demographic section, possible hypertension risk factors section, diet modification, and lifestyle modifications including weight, physical activity, stress, smoking, and medication modification. The first section was designed to get a snapshot of the study population, the second section was designed to collect information regarding the participants’ perception regarding risk factor of hypertension, while the third and fourth sections were designed to gather information about their perception of the needed lifestyle-health related behavior modifications.

Furthermore, this is a community-based study where data were collected using social media. As a result, no ethical approval was needed as no personal identifiable data were collected, and participants were given the opportunity to withdraw at any time. Also, their confidentiality and anonymity were maintained, and they were assured that the data will be presented in an aggregated form. All of the aforementioned was indicated in the cover letter presented at the beginning of the online questionnaire to obtain the participants' consent to participate in the study.

The researchers modified the original questionnaire which then was tested for reliability and validity afterward and used in the study at hand i.e. added different questions in the demographic section and added three questions in two dimensions.

## Results

Overall, the participants showed a high knowledge level in the areas of the three dimensions of our study, namely, hypertension risk factors, diet modifications, and lifestyle modification. However, the respondents were most knowledgeable regarding the first dimension of hypertension risk factors followed by the third dimension, which is lifestyle modification, while the second dimension which is diet modification had the least knowledge level.

Furthermore, in the first dimension, hypertensive patients showed more level of knowledge regarding the risk factors of hypertension more than their non-hypertensive counterparts (i.e. 85% vs 80%, respectively). In the third dimension, hypertensive participants also showed more knowledge than non-hypertensive respondents while almost the same percentage of both showed the same level of knowledge regarding diet modifications.

**Figure 1:**
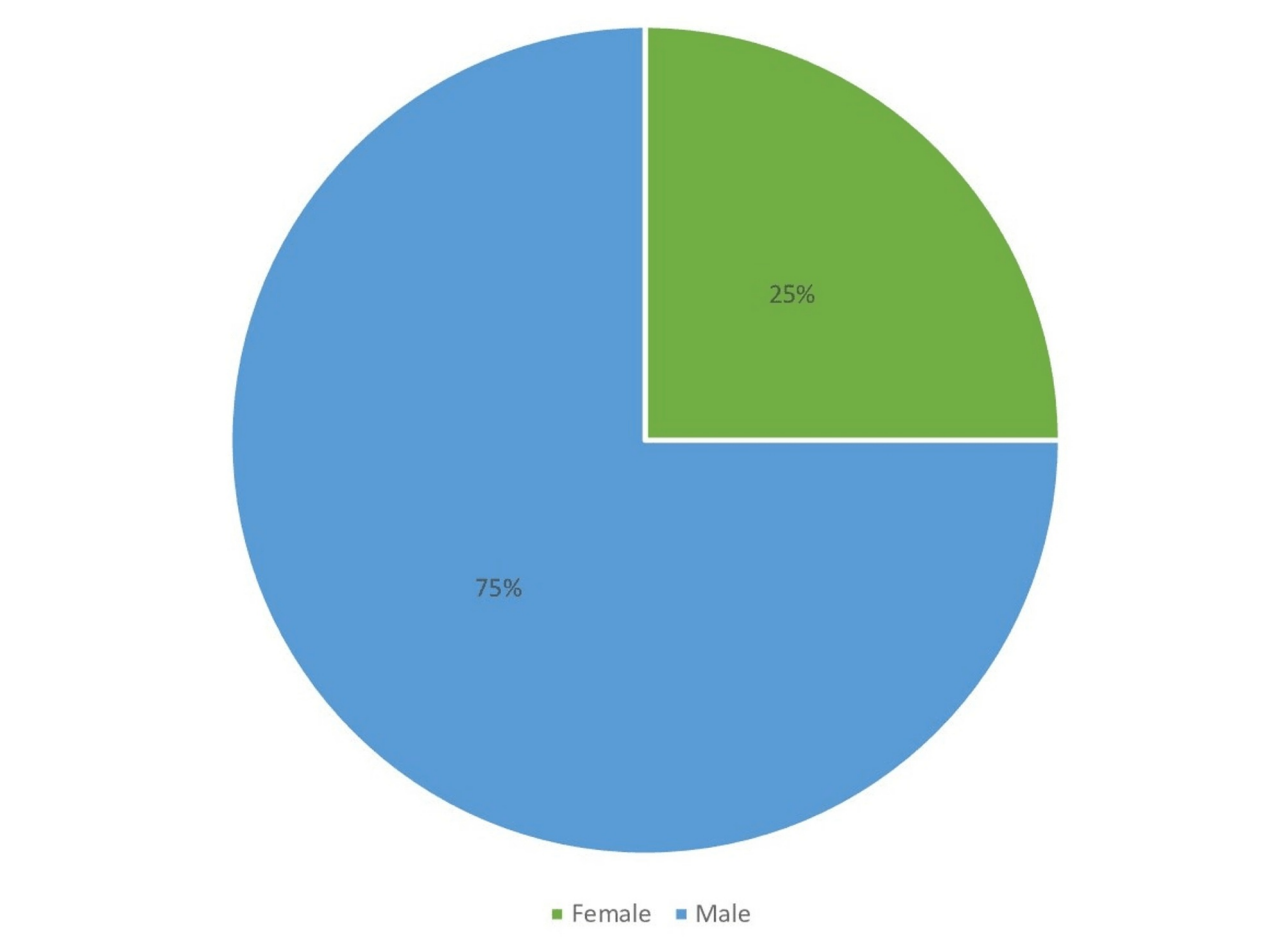
Demographic characteristics of the study participants in terms of gender percentages.

**Figure 2:**
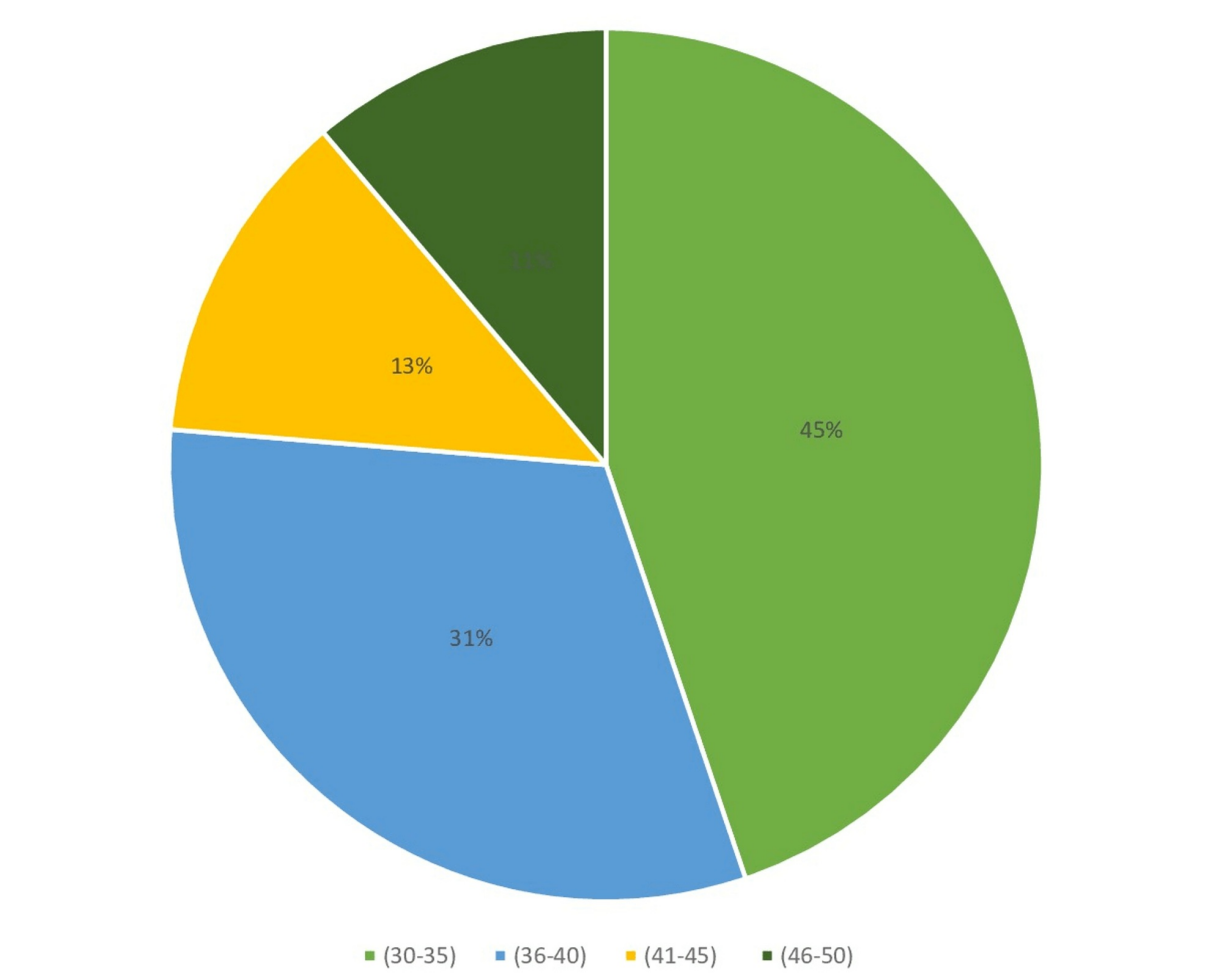
Demographic characteristics of the study participants in terms of age group percentages.

**Figure 3:**
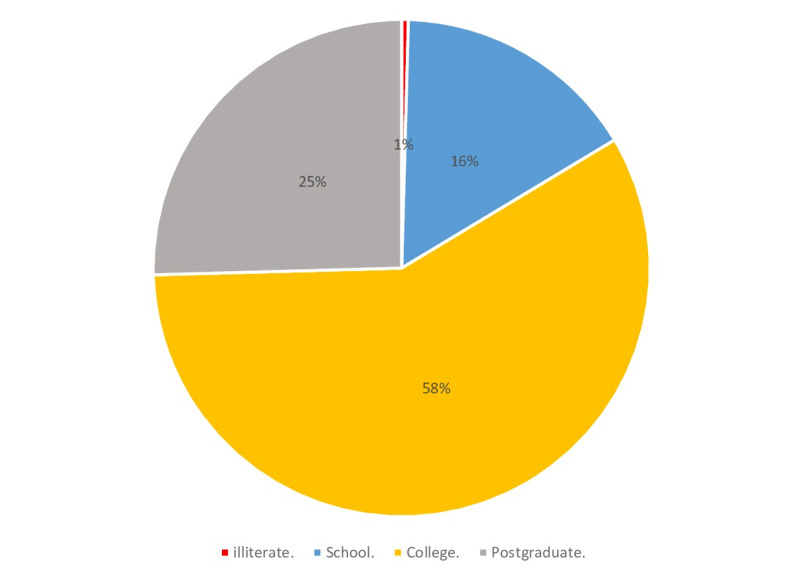
Demographic characteristics of the study participants in terms of educational level percentages.

**Figure 4:**
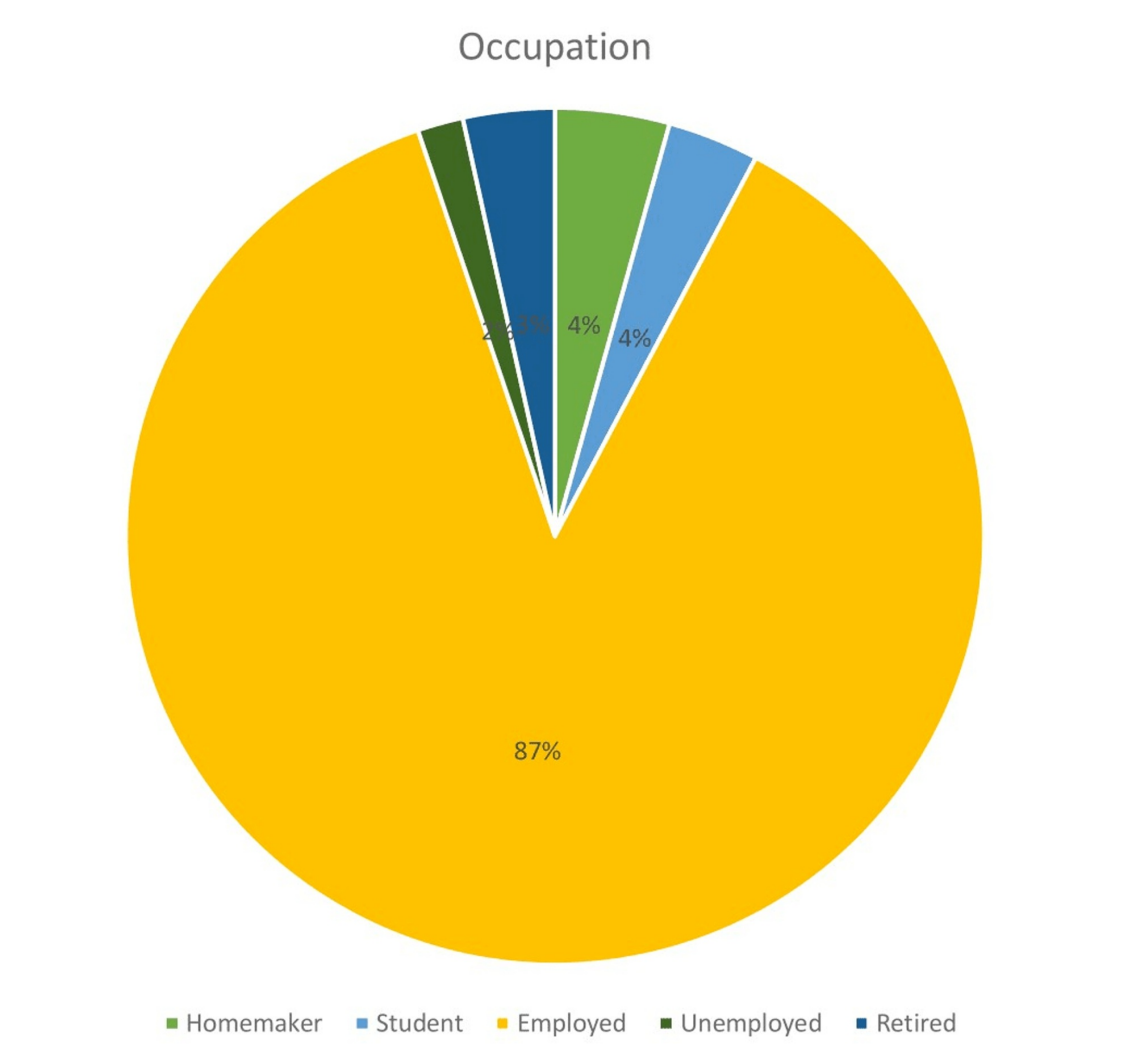
Demographic characteristics of the study participants in terms of occupation percentage - homemaker, student, employed, unemployed, retired.

**Figure 5:**
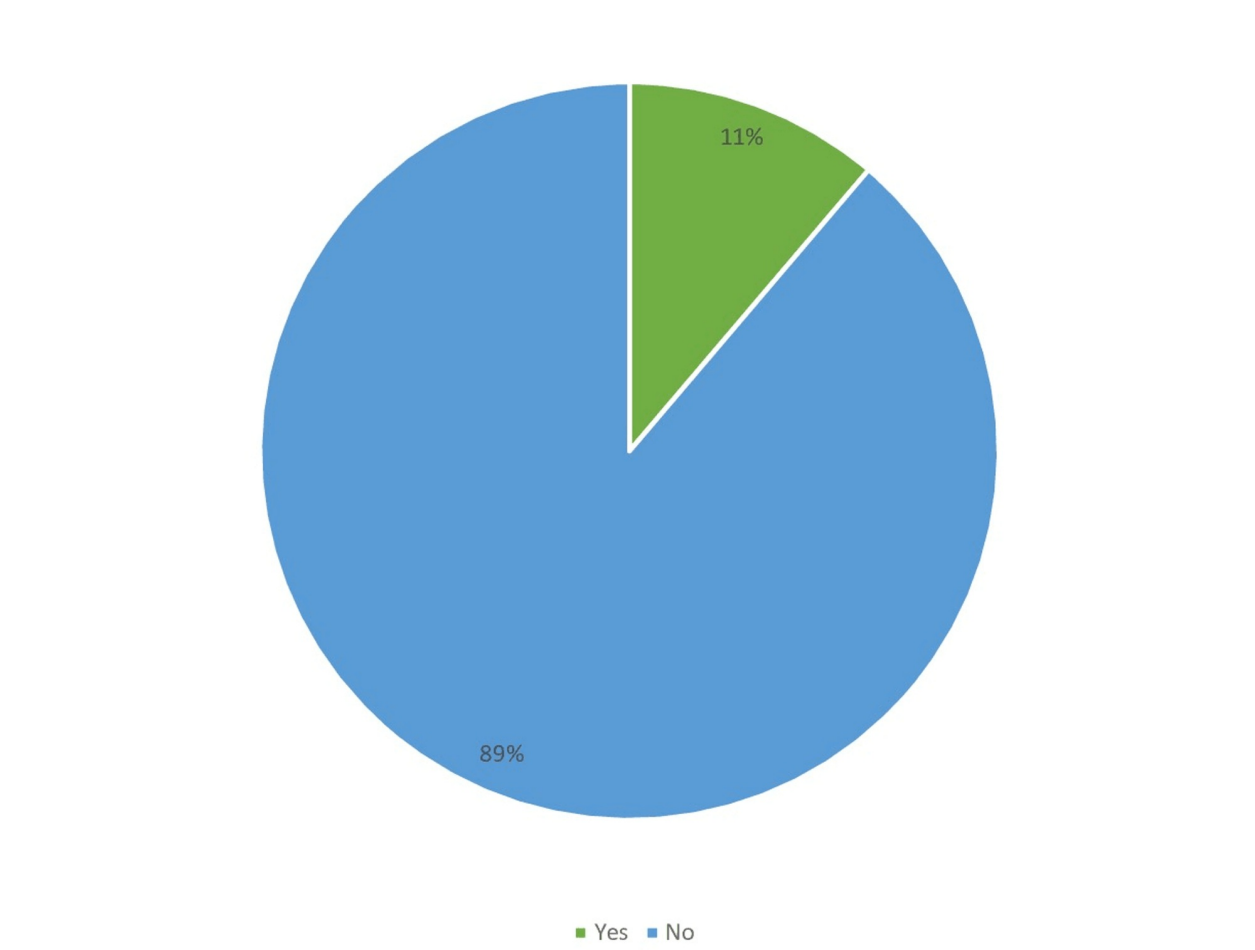
Percentage of study participants with hypertension.

**Figure 6:**
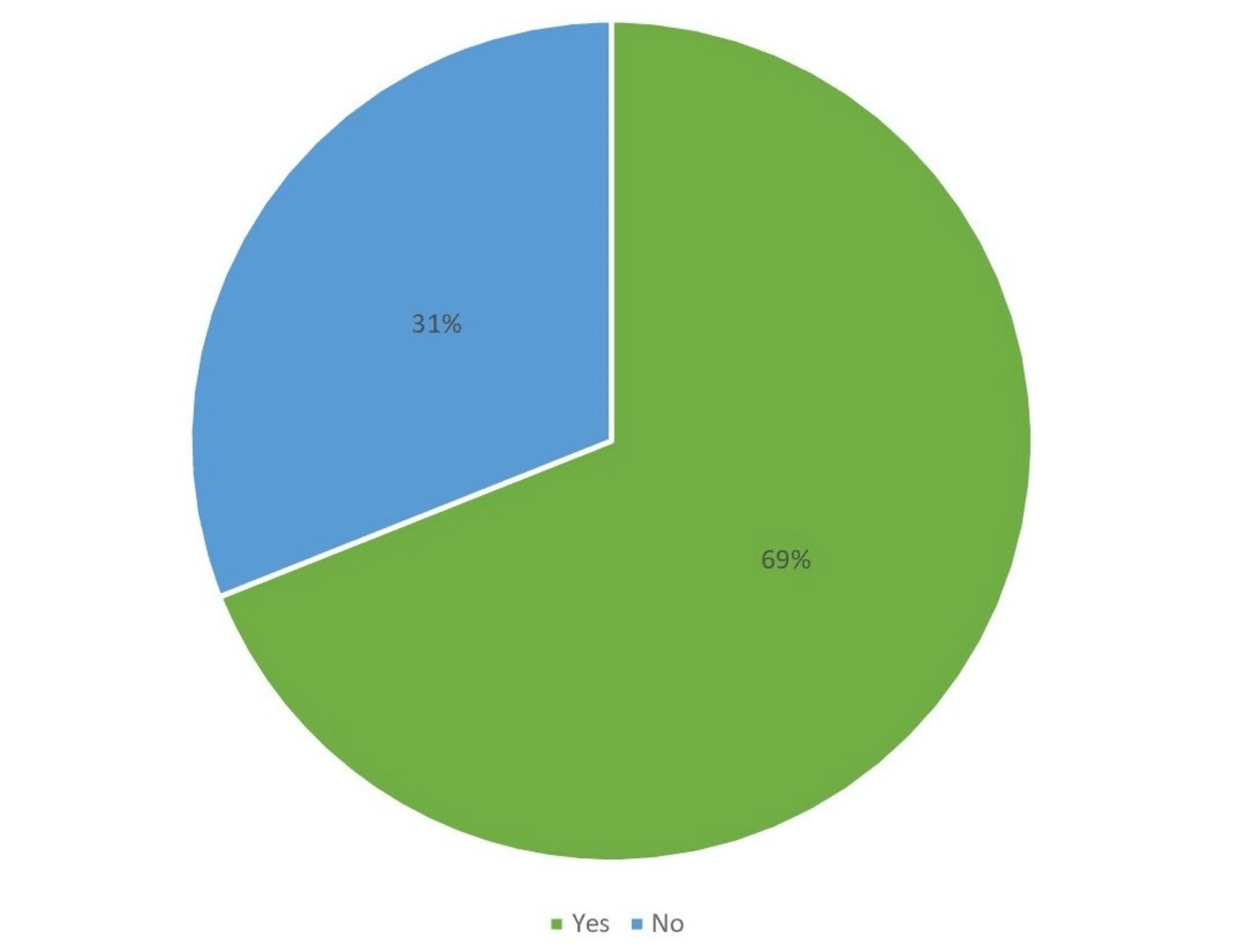
Percentage of study participants with a family history of hypertension.

Figures 1-6 display the distribution of the studied non-hypertensive, and hypertensive patients according to their demographic features. Regarding the gender, Figure 1 shows that 25% of the population were females, while 75% were males. As for the age in Figure 2, it shows that the highest percentages of the population (45%) were in the age group 30-35 years old. Regarding the educational level, and occupation shown in Figures 3-4, the figures reveal that most of the population (87%) are employees, and 58% have a college education. As for the percentage of hypertensive patients and non-hypertensive patients, Figure 5 shows that 11% were hypertensive patients, while 89% were non-hypertensive which can be contributed to their relatively young age. Figure 6 shows that 69% of the population have a positive family history of hypertension. 

**Figure 7:**
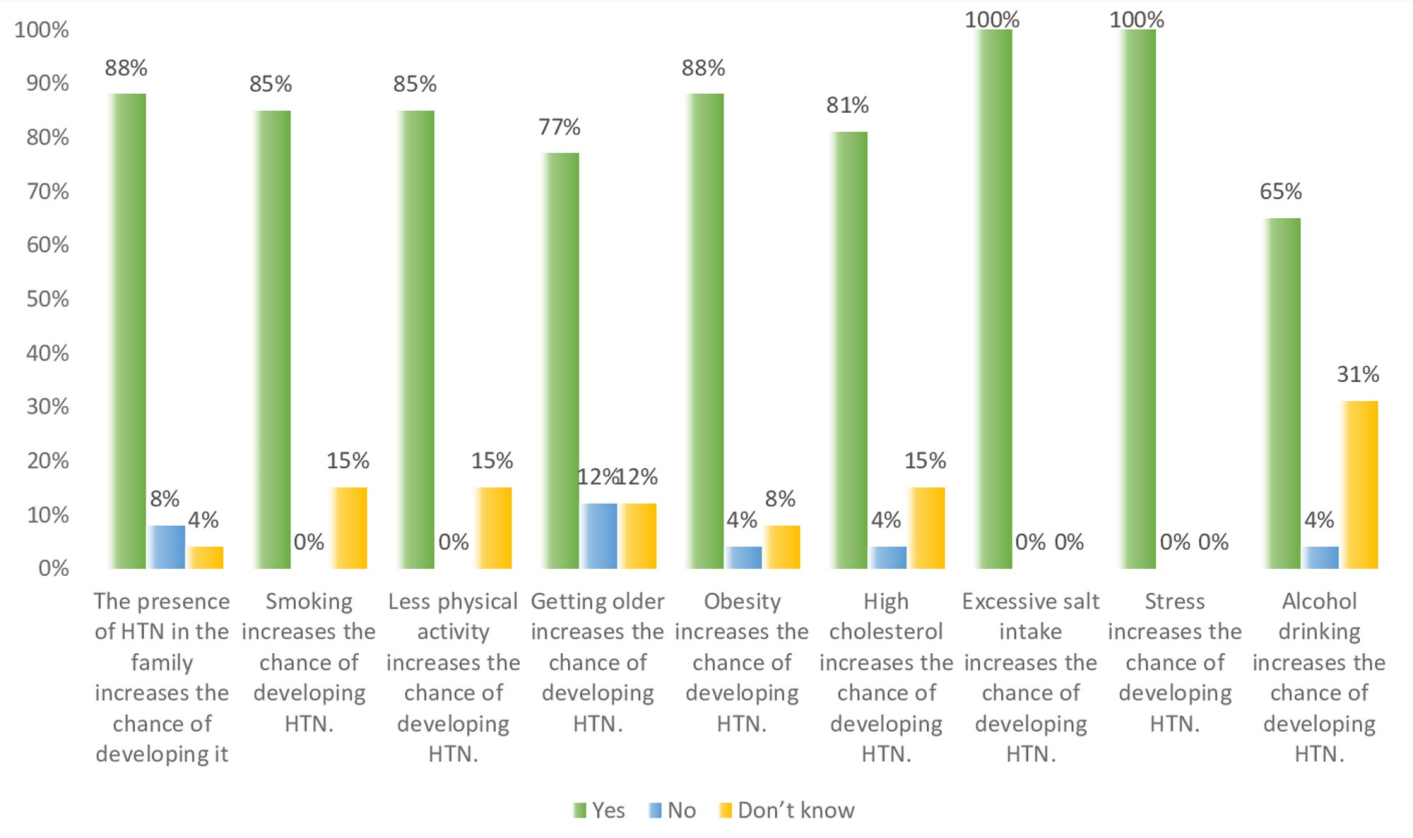
Study participants knowledge level percentage regarding high risk factors of developing hypertension (HTN).

**Figure 8:**
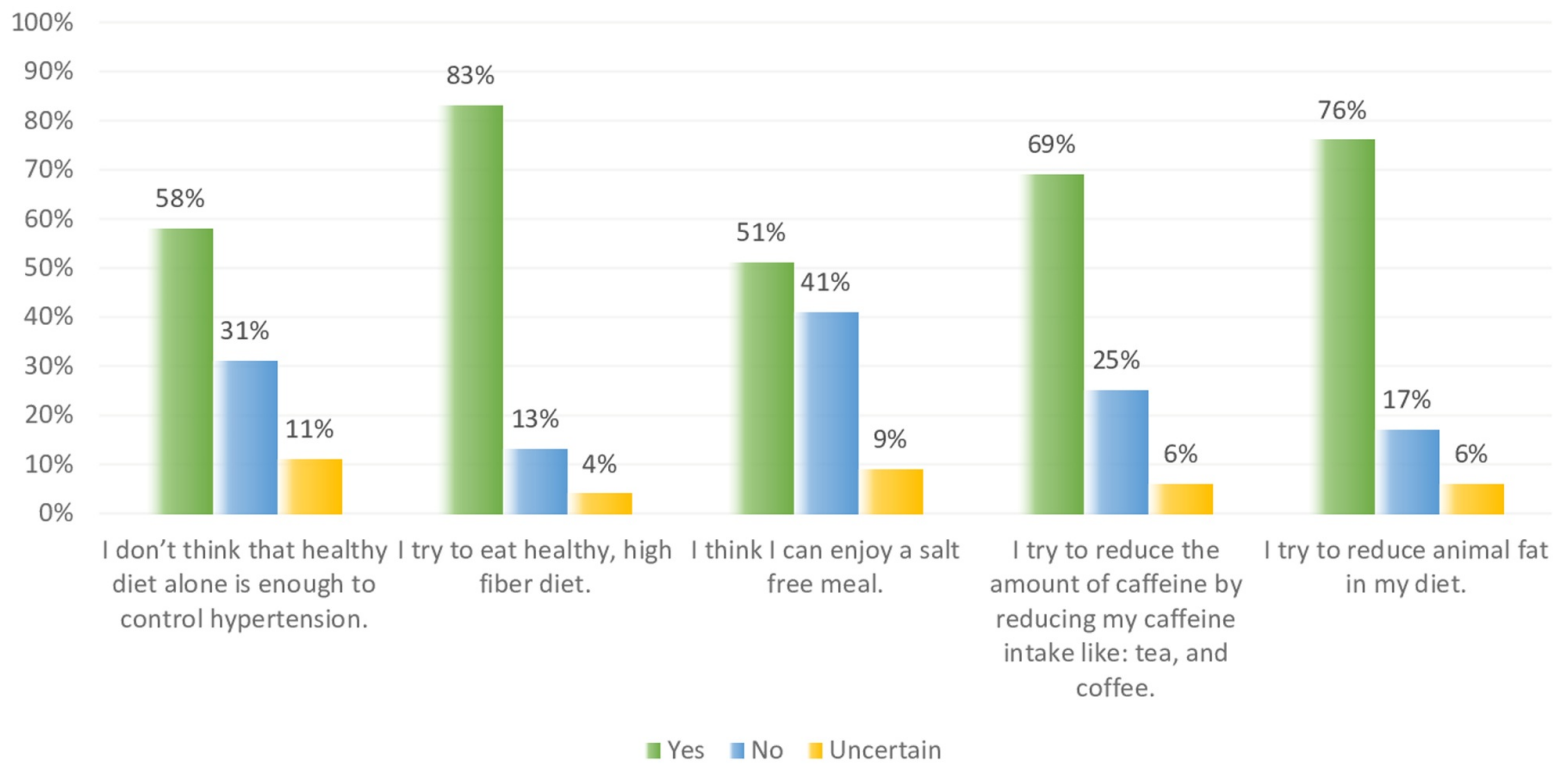
Non-hypertensive, and hypertensive participants’ knowledge level percentage regarding diet modifications.

**Figure 9:**
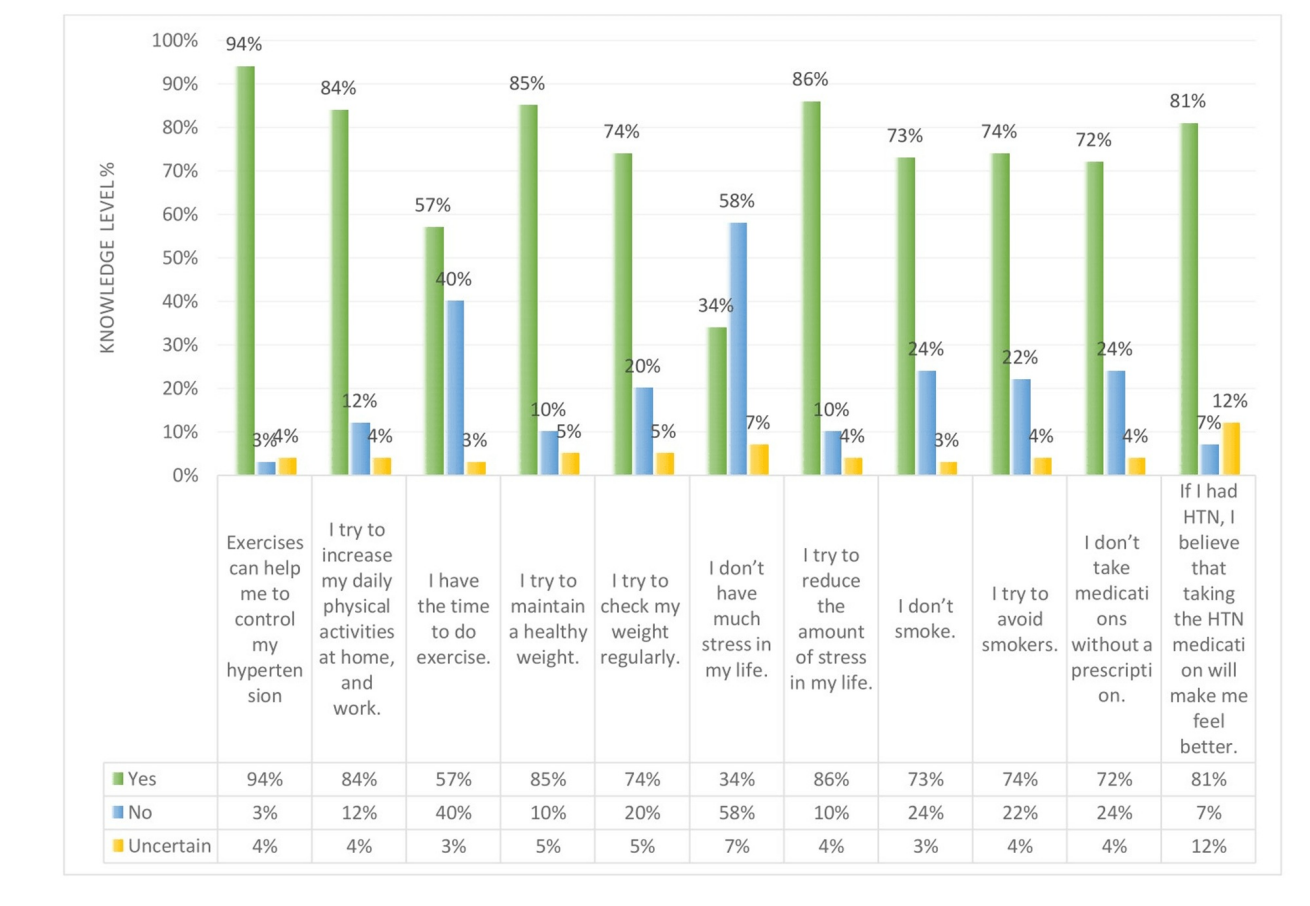
Non-hypertensive, and hypertensive participants’ knowledge level percentage regarding weight and physical activity, stress, smoking, and medication modification. HTN: hypertension

Overall, in Figures 7-9 and Table 1, the results show that both non-hypertensive, and hypertensive patients have well knowledge regarding the risk factors, diet modifications, weight, and physical activity, smoking, and medication modification on hypertension. Figure 7 shows that all the participants are aware that stress and excessive salt intake increase the risk of developing hypertension. On the other hand, some of the hypertensive, and non-hypertensive participants lack the knowledge regarding the effect of alcohol drinking, high cholesterol level, less physical activity, and smoking on increasing the risk of developing hypertension i.e. about 24%, 11%, 10%, and 12%, respectively.

Figure 8 shows the perception of the participants regarding the diet modification. About 83% of participants agree that they try to eat high fiber healthy diet, while 76% and 58% agree that they try to reduce animal fat in their meal, and that healthy diet alone is not efficient to control hypertension. Furthermore, 41% couldn’t enjoy a salt free meal, and 25% didn’t attempt to reduce the amount of their caffeine intake. Figure 9 shows that 94%, and 86% of participants agree that exercise can help to control hypertension, and they try to reduce the amount of stress in their life. On the other hand, 40%, and 58% of participants do not have the time to exercise and have too much stress in their life.

**Table 1:** Non hypertensive, and hypertensive patients’ knowledge regarding risk factors of hypertension, diet modifications, weight and physical activity, stress, smoking, and medication modification.

Items		Sample (232)	
Non hypertensive, and hypertensive patients’ knowledge regarding risk factors of hypertension (HTN)	Yes	No	Don’t know
The presence of HTN in the family increases the chance of developing it.	161	44	27
Getting older increases the chance of developing HTN.	159	50	23
Smoking increases the chance of developing HTN.	186	19	27
Alcohol drinking increases the chance of developing HTN.	149	27	56
Stress increases the chance of developing HTN.	210	13	9
Less physical activity increases the chance of developing HTN.	200	13	19
Obesity increases the chance of developing HTN.	206	9	17
High cholesterol level increases the chance of developing HTN.	195	12	25
Excessive salt intake increases the chance of developing HTN.	216	8	8
Non hypertensive, and hypertensive patients’ Perception regarding diet modifications.	yes	No	Uncertain
I try to eat healthy, high fiber diet.	193	30	9
I don’t think that healthy diet alone is enough to control hypertension.	134	72	26
I think I can enjoy a salt free meal.	118	94	20
I try to reduce the amount of caffeine by reducing my caffeine intake like tea, and coffee.	161	57	14
I try to reduce animal fat in my diet.	177	40	14
Non hypertensive, and hypertensive patients’ Perception regarding weight and physical activity, stress, smoking, and medication modification	Yes	No	Uncertain
exercises can help me to control my hypertension	217	6	9
I have the time to do exercise.	132	92	8
I try to increase my daily physical activities at home, and work.	196	27	9
I try to check my weight regularly.	171	47	11
I try to maintain a healthy weight.	197	24	11
I don’t have much stress in my life.	80	135	17
I try to reduce the amount of stress in my life.	199	24	9
I don’t smoke.	170	56	6
I try to avoid smokers.	171	51	10
I don’t take medications without a prescription.	167	56	9
If I had HTN, I believe that taking the HTN medication will make me feel better.	188	16	28

**Figure 10:**
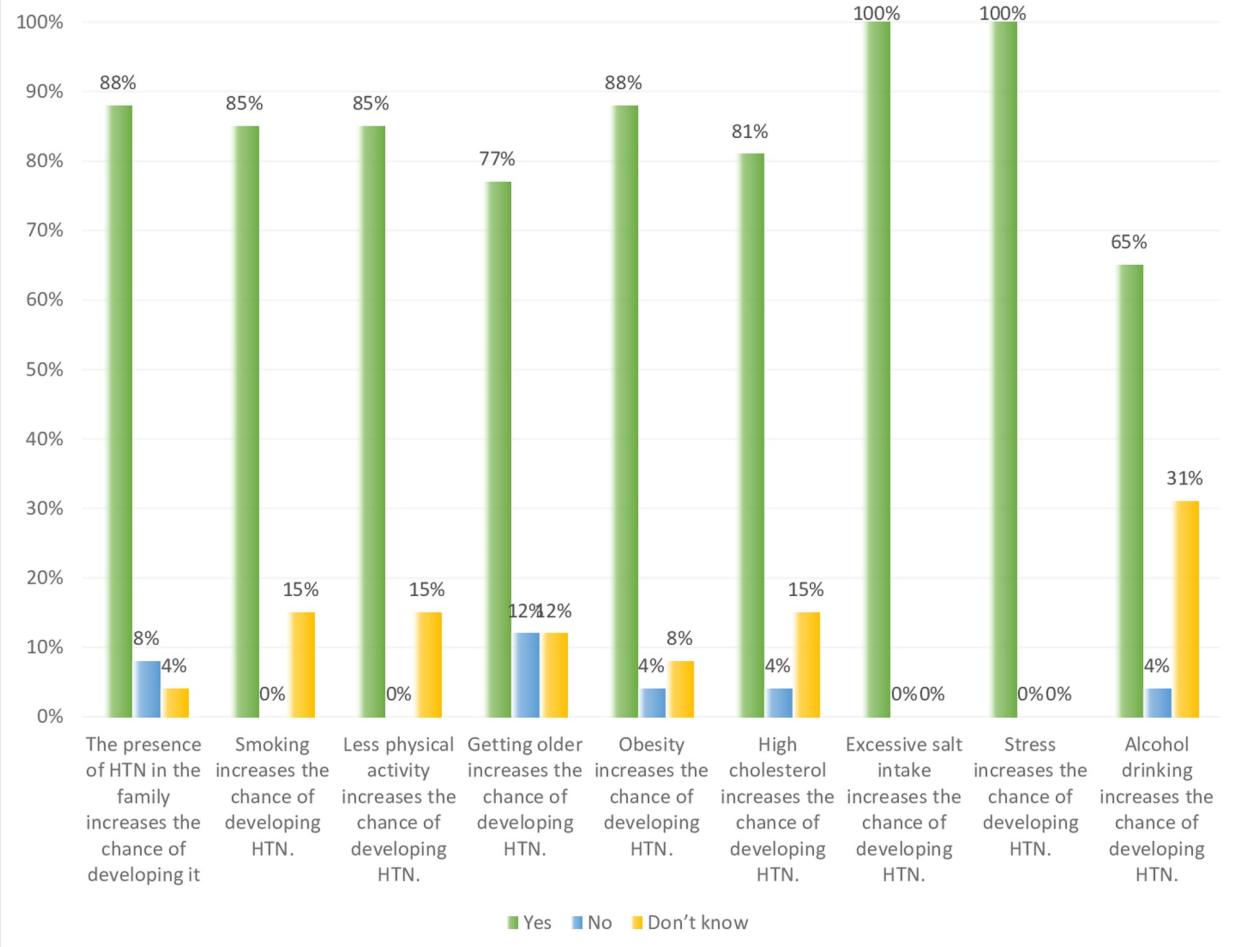
Non-hypertensive, and hypertensive participants’ knowledge level percentage regarding risk factors of hypertension (HTN).

**Figure 11:**
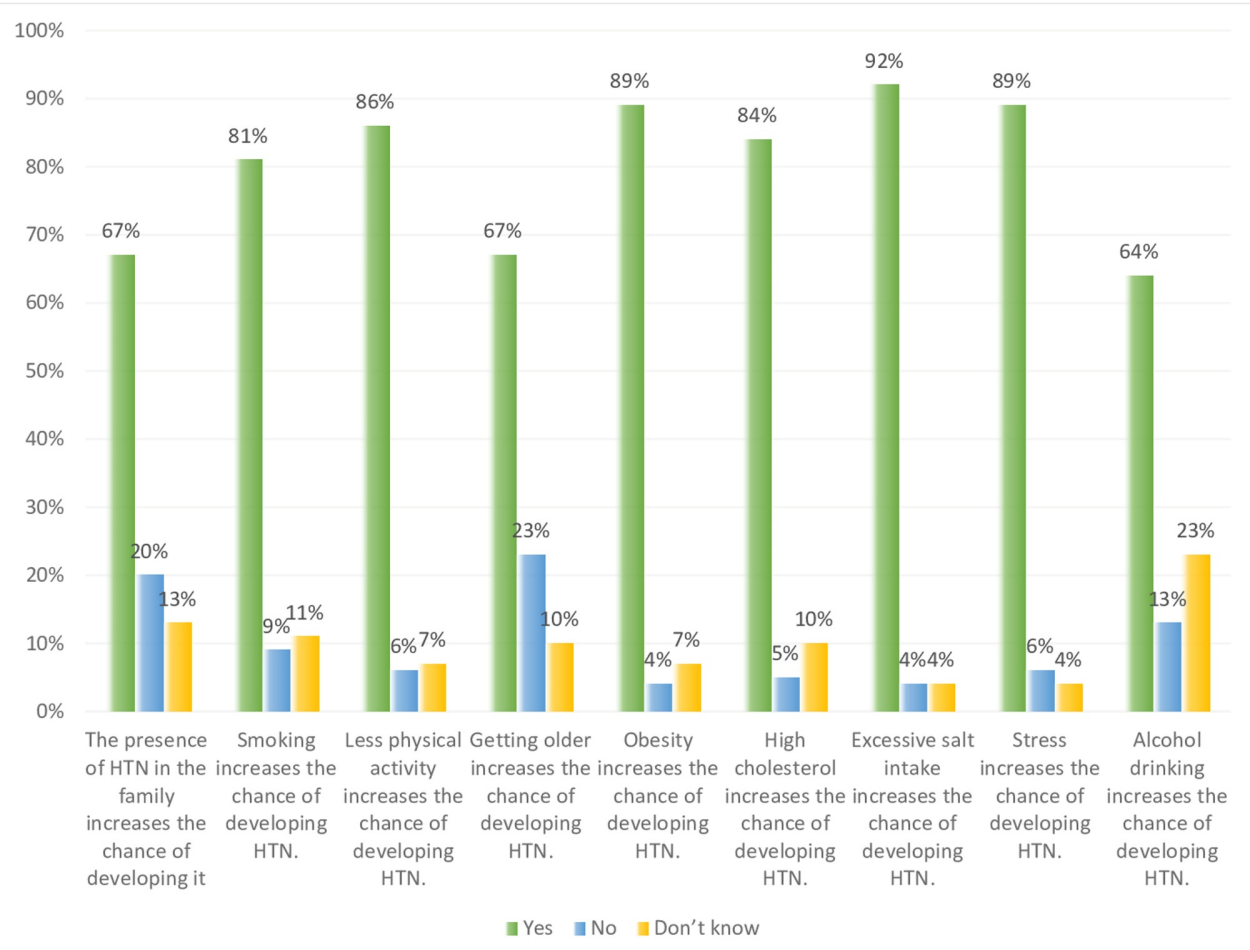
Non-hypertensive, and hypertensive participants’ knowledge level percentage regarding risk factors of hypertension (HTN).

Figures 10-11 shows a comparison between the level of knowledge regarding risk factors of hypertension in non-hypertensive and hypertensive patients. Both hypertensive patients and non-hypertensive individuals have relatively similar levels of knowledge in terms of the risk factors that lead to developing hypertension, namely obesity, less physical activity, and smoking. All hypertensive patients (i.e. 100%) know that excessive salt intake and stress are risk factors while less percentage of non-hypertensive individuals (i.e. 92% and 89%, respectively) believed that these two are hypertension risk factors. For both groups (i.e. hypertensive and non-hypertensive), almost two-thirds of them have knowledge regarding the effect of old age and alcohol intake as risk factors for developing hypertension. Moreover, Figure 10 shows that 88%, 88%, and 85%, respectively of hypertension patients are highly aware that the presence of hypertension in family, obesity, and smoking are risk factors of hypertension. While 31% were not aware of the effect of alcohol drinking on hypertension. Figure 11 shows that 92%, 89%, 89%, and 86% of non-hypertensive participants have high knowledge regarding excessive salt intake, obesity, stress, and less physical activity, respectively are risk factors to develop hypertension. However, 23% and 20% lack the knowledge regarding aging and the presence of hypertension in the family as risk factors for developing hypertension.

**Figure 12:**
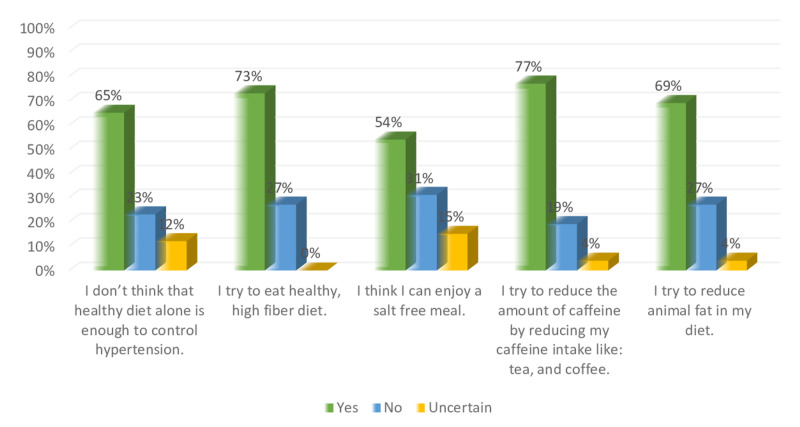
Hypertensive participants’ knowledge level percentage regarding diet modifications.

**Figure 13:**
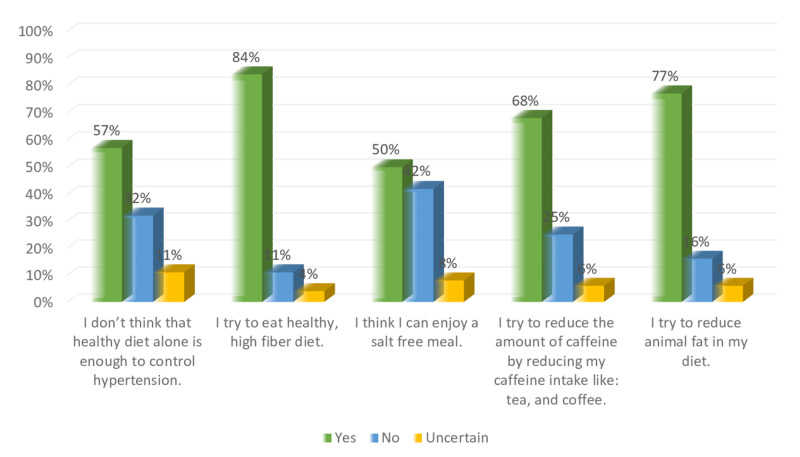
Non-hypertensive participants’ knowledge level percentage regarding diet modifications.

Figures 12-13 show a comparison between the perception of non-hypertensive individuals and hypertensive patients regarding diet modification for hypertension. Both hypertensive patients and non-hypertensive individuals have relatively similar levels of agreement in terms of enjoying a salt-free meal. However, more hypertensive participants were trying to reduce the amount of caffeine intake while more non-hypertensive try to eat a healthy high fiber diet and reduce the amount of animal fat in their diet. More hypertensive participants don not believe that a healthy diet alone is enough to control hypertension compared to their nonhypertensive counterparts. Figure 12 shows that 7% of hypertensive patients try to reduce their caffeine intake, and 73% try to eat a healthy high fiber diet, while 31% cannot enjoy a salt-free meal. On the other hand, Figure 13 shows that 84% of non-hypertensive participants are trying to eat a healthy high fiber diet, and 77% try to reduce animal fat in their diet, while 42% think that they cannot enjoy a salt-free meal.

**Figure 14:**
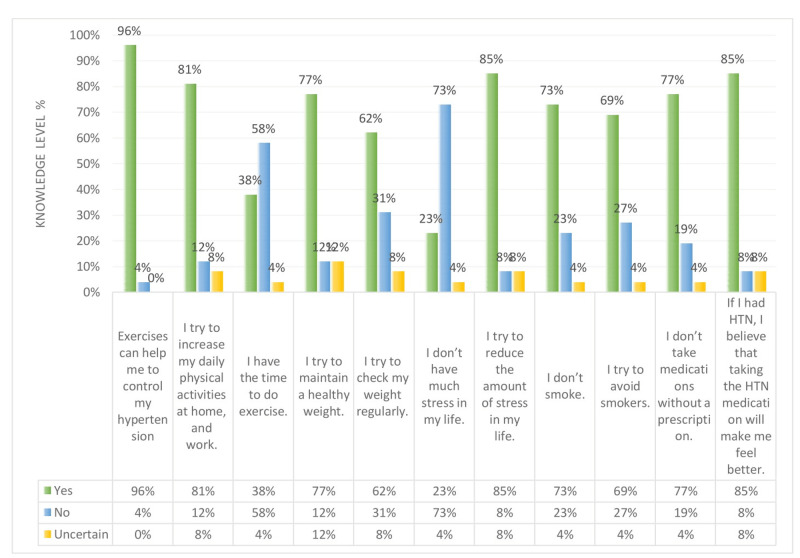
Hypertensive participants’ knowledge level percentage regarding weight and physical activity, stress, smoking, and medication modification. HTN: hypertension

**Figure 15:**
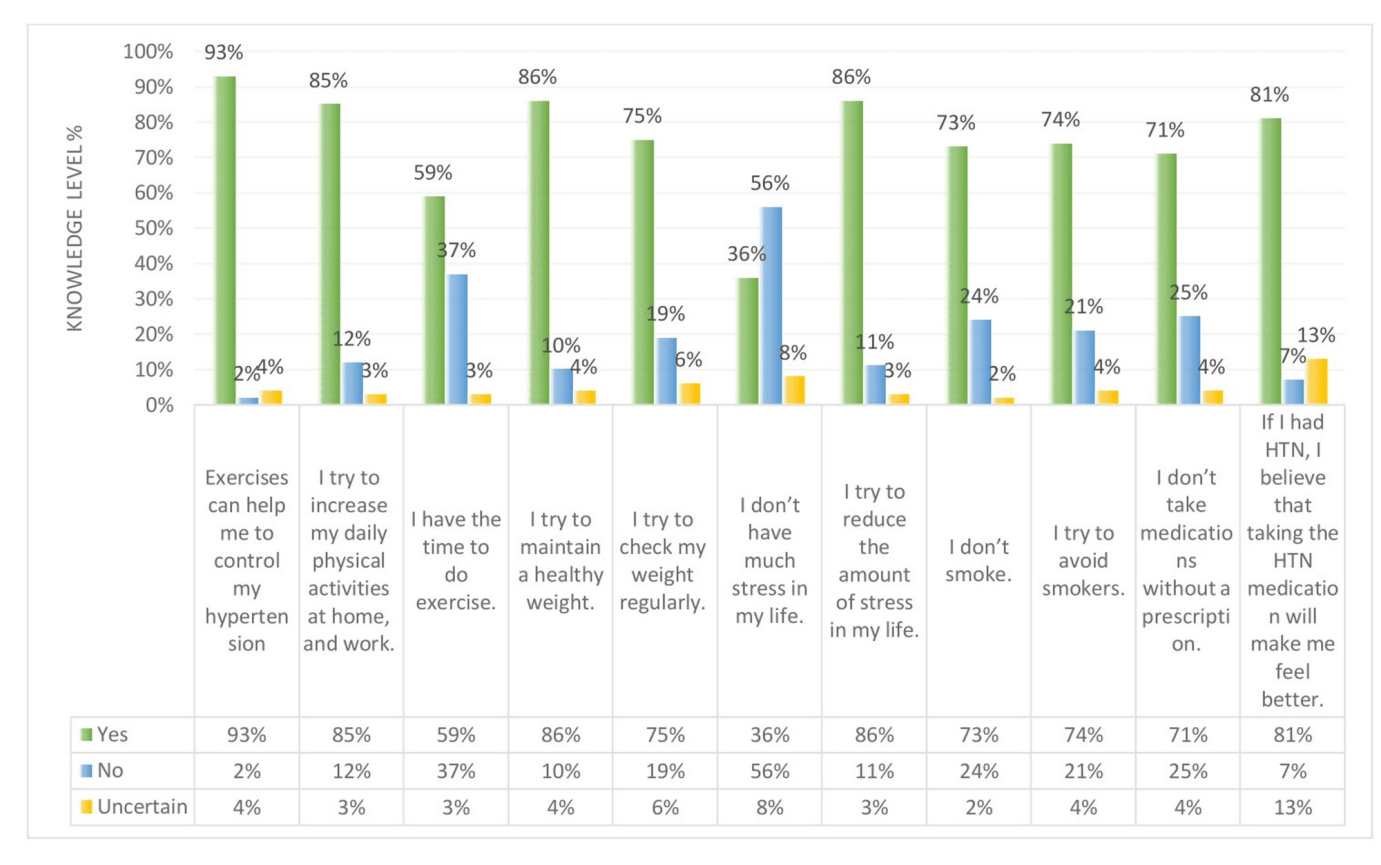
Non-hypertensive participants’ knowledge level percentage regarding weight and physical activity, stress, smoking, and medication modification. HTN: hypertension

Figures 14-15 show a comparison between the perception regarding lifestyle modifications like weight, physical activity, stress, smoking, and medication modification in non-hypertensive participants and hypertensive respondents. Figure 14 explains that 96% of hypertensive patients believe that exercising can help them control their hypertension. However, 58% feel that they do not have the time to exercise. Regarding the use of hypertension medication, 85% of hypertensive patients feel better after taking the medication. As for stress, 73% are considered to have a high level of stress in their life. At the same time, 85% try to reduce the amount of stress in their life. On the other hand, Figure 15 shows that non-hypertensive participants also have a high level of stress in their life (i.e. 56%), and more than half try to reduce the amount of stress in their life. Regarding physical activity, 85% of non-hypertensive participants try to increase their daily activities at home and work.

Finally, both groups have the same knowledge level that does not differ significantly. Gender is not a factor of significance for hypertension, both male and female groups do not statistically differ (p=0.471), but a family history of hypertension shows a significant relationship among the two groups (p<0.006). Nevertheless, age, education, and occupation do not relate significantly among both hypertensive and non-hypertensive individuals.

## Discussion

Hypertension is one of the most common health challenges worldwide due to its high morbidity and mortality rate [12]. Therefore, it is important to promote awareness and knowledge within the community to prevent developing hypertension and related cardiovascular diseases [7]. Thus, the first step is to measure the community level of knowledge and their perception regarding the disease as to whether they were hypertensive or not. This study was conducted to determine the perception, and knowledge of non-hypertensive and hypertensive individuals regarding hypertension. In our research, we found out that all of the literature reviewed was concerned with the perception of hypertensive patients only which limits our ability to compare our results for non-hypertensive individuals to the existing literature.

Our study revealed that the knowledge regarding the risk factors of hypertension was (85%). The perception that hypertension can be controlled by medication was among 81% of the participants (which is one of the questions of the third dimension of lifestyle modifications); this was in agreement with Azubuike and Kurmi's study [13] and Kongarasan and Shah's study [14], while it disagrees with the results of the study by Okwuonu et al. [15]. In their study, Azubuike and Kurmi found out that there was a fair level of knowledge in hypertensive patients regarding hypertension. Furthermore, the study of Kongarasan and Shah which was done in India to assess the awareness of hypertension among hypertensive patients revealed that there was a high prevalence of knowledge regarding the risk factors of hypertension (94%), and a high percentage of them (90%) agreed that hypertension can be controlled by medications which were consistent with our results. On the other hand, our results disagree with the findings of the study by Okwuonu et al. [15] which stated that the level of awareness and practice of lifestyle modification of hypertensive patients in order to control hypertension was poor.

In addition, our study results showed that more than half of both hypertensive and non-hypertensive participants were aware of the role of positive family history of hypertension, smoking, and high cholesterol level in increasing the risk of developing hypertension. As for the level of non-hypertensive and hypertensive patients' perception related to lifestyle modifications, our study results showed that most of the non-hypertensive and hypertensive patients have very good perceptions regarding lifestyle modifications: smoke, weight, and physical activity. While 40% and 58% have difficulty in finding the time to exercise and have a high level of stress in their life (Figure 9). This result was inconsistent with Kofi’s (2011) study [16], and the study by Bhandari et al. (2012) [17] while it disagrees with the results of the study by Awotidebe (2014) [18]. The study by Kofi revealed that 97% of participants agree that modification of lifestyle behaviors could be associated with preventing and controlling hypertension [16]. Furthermore, the study by Bhandari et al. shows that more than 50% of hypertensive patients have a high level of knowledge regarding controlling hypertension [17]. On the other hand, our results disagree with the findings of the study by Awotidebe et al. which revealed that the majority had poor knowledge of exercise for hypertension control [18].

Our study also shows that 69% of the population whether hypertensive or not, have a positive family history of hypertension, which may have contributed to their high level of knowledge regarding the disease. In addition, the high level of education (with a college degree) might also have caused their good perception regarding the hypertensive risk factors and life modifications needed to either prevent or control it. The revealed interest in improving the quality of their lifestyle of the participants may have also contributed to their high level of knowledge about hypertension. Our results indicated that both groups have the same knowledge level that does not differ significantly, where gender is not a factor of significance for hypertension-related knowledge level. In addition, age, education, and occupation do not relate significantly among both hypertensive and non-hypertensive individuals. Yet, a family history of hypertension shows a significant relationship between the two groups.

## Conclusions

Overall, we found that both groups have adequate knowledge regarding risk factors, diet modifications, weight, physical activity, stress, smoking, and medication modification. A family history of hypertension appeared to has a significant relationship among the two groups. However, both groups reported that they have difficulty finding the time to exercise as well as experiencing stress in their life, yet, they reported that they are trying to reduce it. The results of high knowledge among the respondents might be contributed to the participants' high educational level as well as the fact that a lot of them have a family history of hypertension.
